# The Potential Role of Iron and Copper in Pediatric Obesity and Nonalcoholic Fatty Liver Disease

**DOI:** 10.1155/2015/287401

**Published:** 2015-07-26

**Authors:** Alexandra Feldman, Elmar Aigner, Daniel Weghuber, Katharina Paulmichl

**Affiliations:** ^1^First Department of Medicine, Paracelsus Medical University, Müllner Hauptstrasse 48, 5020 Salzburg, Austria; ^2^Obesity Research Unit, Paracelsus Medical University, Müllner Hauptstrasse 48, 5020 Salzburg, Austria; ^3^Department of Pediatrics, Paracelsus Medical University, Müllner Hauptstrasse 48, 5020 Salzburg, Austria

## Abstract

Obesity is a rapidly growing health problem and is paralleled by a multitude of comorbidities, including nonalcoholic fatty liver disease (NAFLD). NAFLD has become the most common chronic liver disease in both adults and children. The current understanding of NAFLD is still fragmentary. While simple steatosis is characterized by the interplay between excessive free fatty acid accumulation and hepatic insulin resistance, the progression to NASH has been related to oxidative stress and a proinflammatory state with dysbalanced adipokine, cytokine levels, and endotoxin-mediated immune response. In addition, oxidative stress has been suggested to play a central role for the sequelae leading to NASH. Trace elements are critical in regulatory, immunologic, and antioxidant functions resulting in protection against inflammation and peroxidation and consequently against the known comorbidities of obesity. Disruptions of the metal detoxification processes located in the liver are plausibly related to NAFLD development via oxidative stress. Perturbations of iron and copper (Cu) homeostasis have been shown to contribute to the pathogenesis of NAFLD. This review presents current data from pediatric studies. In addition, data from adult studies are summarized where clinical relevance may be extrapolated to pediatric obesity and NAFLD.

## 1. Introduction

Obesity is a rapidly growing health problem. The increase in the prevalence of obesity is paralleled by a multitude of comorbidities, including nonalcoholic fatty liver disease (NAFLD) [1]. The latter encompasses a range of clinicopathological entities ranging from simple steatosis through nonalcoholic steatohepatitis (NASH) to cirrhosis and end-stage liver disease. NAFLD has become the most common chronic liver disease in both adults and children. Schwimmer et al. showed in a landmark US autopsy study that fatty liver prevalence increases with age, ranging from 0.7% for ages 2 to 4 up to 17.3% for ages 15 to 19 years, the highest rate being seen in obese children (38%) [2]. The current understanding of the pathogenetic mechanisms behind NAFLD and its phenotypic variations is still fragmentary. Although pediatric NAFLD has been shown to be relatively benign in the majority of patients, severe hepatic complications including cirrhosis and the need for liver transplantation already occur in a subgroup of children with obesity [3]. Histopathologically, advanced fibrosis, which includes bridging fibrosis and cirrhosis, is seen as the most important factor in determining the prognosis of NAFLD. Children with NASH have been shown to present with distinct histopathological subtypes. Key differences between these subtypes include age, sex, race/ethnicity, and severity of obesity [4]. Liver biopsy thus remains the gold standard for assessing NAFLD severity and staging of fibrosis [5]. However, the biological underpinnings of these subtypes remain to be elucidated.

Besides calorie excess, perturbations of iron and Cu (Cp) homeostasis have been shown to contribute to the pathogenesis of NAFLD, although investigations have mainly been conducted in adults. This review summarizes available data from pediatric studies. In addition, data from adult studies are summarized where clinical relevance may be extrapolated to pediatric obesity and NAFLD.

## 2. The Physiology of Iron

Iron is mainly required for heme biosynthesis in erythropoiesis [6]. Approximately 1-2 mg of iron is absorbed from the duodenum daily [7] whereas the majority is obtained by the reuse of senescent erythrocytes.

Dietary iron is absorbed via the divalent metal transporter 1 (DMT1) as Fe^2+^ in the proximal duodenum [8]; subsequently the transport of iron trough the basolateral membrane is performed by ferroportin (FPN) [9]. Although heme constitutes an important source of iron from the diet and also supplies iron for cellular iron requirements via reutilization, the mechanism for enteral heme uptake has not yet been identified [10]. Prior to entering the bloodstream loaded onto transferrin [11], iron undergoes oxidation into the ferric form (Fe^3+^) by the transmembrane Cu-dependent ferroxidase hephaestin [12].

Cells generally facilitate the uptake of iron via the transferrin receptor (TfR1) according to intracellular iron demand [13] and export iron via FPN. Excess iron is mainly stored in hepatocytes or macrophages as ferritin [14].

Systemic iron homeostasis is maintained in a hormone-like negative feedback mechanism by the 25-amino acid peptide hormone hepcidin (hepatic bactericidal protein) [15]. Hepcidin exerts its regulatory functions on iron homeostasis via binding to FPN, thereby leading to FPN phosphorylation, degradation, and consequently blockage of cellular iron export which induces a decrease in serum iron [16]. Apart from the liver, hepcidin is produced by adipose tissue (AT), macrophages, and pancreatic islet cells [17, 18]. The expression of hepcidin is stimulated by iron, hypoxia, and proinflammatory cytokines or adipokines [19], such as interleukin 6 (IL-6) and also leptin [20, 21]. Hepcidin deficiency leads to uninhibited iron uptake and is the pathophysiological mechanism underlying hereditary hemochromatosis [22]. Conversely, hepcidin expression is enhanced in inflammatory conditions leading to iron retention in macrophages and decreased iron uptake from enterocytes, as observed in the anemia of chronic disease [23].

## 3. Iron Status in Obese Children

Regarding dietary iron intake no difference has been found between obese and nonobese children [24, 25]. However the prevalence of iron deficiency was higher in obese children compared to normal weight subjects in industrialized countries [26, 27]. Both serum iron concentrations and iron stores, as indicated by serum ferritin concentrations, show a negative correlation with BMI [28, 29]. Likewise, iron deficiency increased with the percentage of body fat and visceral fat mass in preadolescents [25]. The prevalence of iron deficiency is similar in obese pubertal males and females [28]. This is in contrast to normal weight children, where iron deficiency is more prevalent in girls than boys [30].

In addition to the higher prevalence of iron deficiency in obese adolescents, iron supplementation is less effective in overweight children due to decreased duodenal iron absorption compared to normal weight peers which also may be explained by increased circulating hepcidin [31]. Along this line, weight reduction leads to a decrease of hepcidin and leptin levels and further to an increase of iron absorption and an improvement of iron status, thus providing indirect evidence that obesity-associated inflammation underlies insufficient duodenal iron uptake [32, 33].

## 4. Mechanisms Underlying Iron Deficiency in Juvenile Obesity

A study by Aeberli showed that no difference occurs either in nutritional iron intake or in dietary iron bioavailability between obese and nonobese children [34]. However lower iron absorption has consistently been observed in overweight subjects [35]. These findings suggest that the diminished iron uptake via enterocytes can be viewed as main cause for iron dysregulation in obesity.

Hepcidin is the key regulator of iron metabolism and hence has been investigated in adolescent obesity. Iron deficiency in overweight children is associated with elevated serum hepcidin concentrations [34]. Several studies have found significantly higher concentrations of hepcidin in overweight children in comparison to normal weight children [34, 36]. This may be caused by obesity related inflammation since proinflammatory cytokines enhance hepcidin expression [37] and are increased in obese children [34].

**Figure 1:**
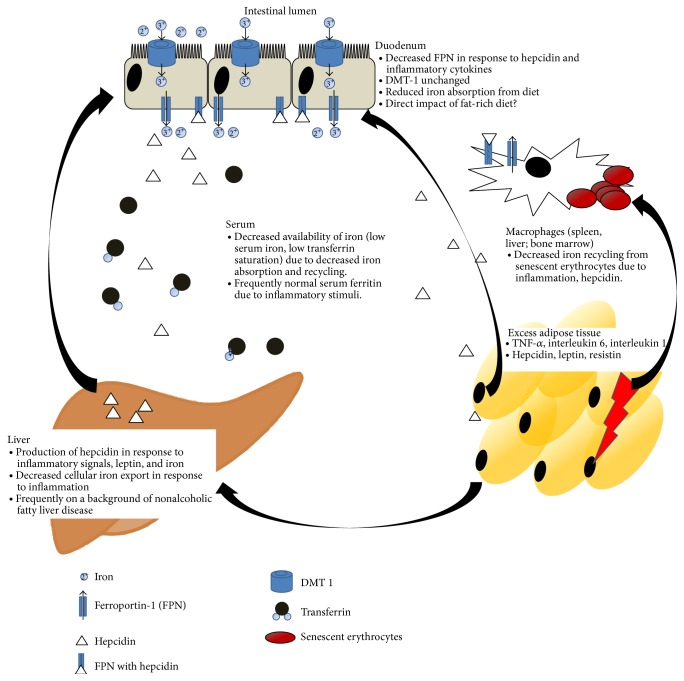
Current understanding of molecular links between obesity and iron deficiency. Obese adipose tissue is characterized by an increased production of several proinflammatory cytokines and adipokines as opposed to healthy lean adipose tissue. These may directly impact iron absorption from the enterocyte. Additionally, proinflammatory cytokines such as interleukin 1 and interleukin 6 represent potent inducers of hepcidin production in the liver, which may further impair iron absorption. Both cytokines and hepcidin lead to iron retention in spleen, liver, or bone marrow macrophages, thereby lowering serum iron concentrations and iron availability for erythropoiesis.

Weight loss in obese children leads to a decrease of serum hepcidin levels along with improvement of iron absorption [32]. Although in quantitative terms hepcidin is mainly derived from the liver, AT of morbidly obese, anemic, iron deficient subjects produces hepcidin, in contrast to AT of lean subjects [17]. Hence, hepcidin produced in obese AT may directly impact iron homeostasis. However, a recent study shows that hepcidin expressed in AT may not be secreted and thus may have no effect on systemic iron homeostasis [38]. Still, AT-derived cytokines such as IL-6 and IL-1 function as potent inducers of hepcidin expression in the liver also in obesity, thereby contributing to elevated serum hepcidin concentrations proportional to AT inflammation [39]. The current understanding of mechanisms underlying iron deficiency in obesity is summarized in Figure 1.

## 5. Iron and Pediatric NAFLD

Although juvenile obesity is linked to a higher prevalence of iron deficiency, in pediatric NAFLD serum ferritin concentrations are within normal range [40]. Additionally, transferrin saturation, an early indicator of increasing iron stores, is higher in obese children with NAFLD compared to those without. According to Demircioğlu et al. these observations may represent incipient iron perturbations corresponding to the dysmetabolic iron overload syndrome (DIOS) in adults [41]. Hence, NAFLD may impact on iron homeostasis in a way that differs from mere obesity related inflammation leading to iron deficiency. DIOS is characterized by elevated serum ferritin concentrations with normal or mildly transferrin saturation in patients with NAFLD or various compounds of metabolic syndrome, particularly in males or postmenopausal women [42, 43]. Similar to these data from adult populations, 22% of children with NAFLD had mild siderosis on histological examination. The hepatic iron deposition in children is predominantly found in nonparenchymal Kupffer cells [40]. In contrast to siderosis in adults with NAFLD, no association between the pattern of iron accumulation and severity of fibrosis or insulin resistance has been found in children [40, 44]. Along the same line of evidence, higher iron stores were related to serum alanine amino transferase (ALT) and metabolic parameters such as lipids and fasting glucose in otherwise healthy teenagers, which was likewise reminiscent of the link of elevated iron stores with insulin resistance in adults [45]. Moreover, children with NAFLD have higher serum levels of hepcidin in comparison with obese children without NAFLD which may reflect higher iron stores or inflammation [41]. In adults, excess iron is assumed to enhance endoplasmatic reticulum [46] and oxidative stress in NAFLD, and thereby liver damage, due the formation of highly toxic hydroxyl radicals via the Fenton reaction [47]. Conversely, a recent study by Moya et al. reported that biopsy proved NASH patients of 12 to 20 years had significantly lower serum iron concentrations and undetectable histological iron compared to controls and to subjects with simple steatosis. Furthermore, an elevated expression of transferrin receptor 2 was found, which is a well-recognized positive upstream regulator of hepcidin expression [48].

In addition, while simple steatosis is characterized by the interplay between excessive free fatty acid accumulation and hepatic insulin resistance, the progression to NASH has been related to oxidative stress and a proinflammatory state with dysbalanced adipokine, cytokine levels, and endotoxin-mediated immune response, among other factors [49]. In particular, oxidative stress has been suggested to play a central role for the sequelae leading to NASH. Studies in both human livers and an animal model of NASH have demonstrated that the increased expression of the cytochrome P450 (CYP 2E1) and reactive oxygen species (ROS) are related to FFA levels as well as mitochondrial dysfunctions [50]. Disruptions of the metal detoxification processes located in the liver are plausibly related to NAFLD development via oxidative stress [51, 52].

In adults, several studies demonstrated that hyperferritinemia and/or iron deposition in liver biopsies were linked to more progressed stages of NAFLD, insulin resistance and that it may even be related to the development of hepatocellular carcinoma in NASH [53, 54], the mortality of patients on the transplantation waiting list, and it also had an impact on posttransplant mortality [55, 56]. Thus the prevailing body of evidence suggests that excess iron is a contributing factor for the progression of steatosis to NASH, liver cirrhosis in adults, but these aspects have not been examined sufficiently in pediatric cohorts. The natural course, the long-term outcome, and the onset of these pathophysiological changes in children and adolescents remain to be studied in detail, in order to shed light on the role of iron in the transition of adolescent to adult NAFLD.

## 6. Copper and Pediatric NAFLD

A sufficient supply of Cu is known to be essential for many physiologic processes, as chronic Cu deficiency is associated with anemia, leucopenia, myelopathy, skin pathologies, and dysfunctional lipid metabolism [57, 58]. We could previously show that reduced hepatic Cu concentrations are found in human NAFLD and are associated with more pronounced hepatic steatosis, NASH, and components of the metabolic syndrome. In addition, a Cu-restricted diet induced hepatic steatosis and insulin resistance in Sprague-Dawley rats [59]. Population based investigations suggested that Cu deficiency may be associated with atherogenic dyslipidemia [60]. Furthermore, in rodent models Cu restriction leads to hypertension, elevated triglycerides and cholesterol, and modified lipoprotein composition [58, 61]. Oxidative stress is involved in the pathophysiology of NAFLD and Cu/Zinc superoxide dismutase which neutralizes oxidative stress and requires Cu for its biological function, potentially linking Cu and antioxidant defence [62]. Correspondingly, a Cu deficient diet was linked to an enhanced proinflammatory response in Sprague-Dawley rats [63]. Changes in mitochondrial morphology and function have been reported in human NAFLD and similar changes are found as a consequence of Cu deficiency [64]. Hence, these pathophysiological mechanisms may represent links between Cu homeostasis and NAFLD.

A recent study showed that trace element levels in obese children may vary strongly due to poor nutritional status [65]. In pediatric cohorts, serum Cu levels were reported to be significantly higher in obese patients than in normal weight controls in some [66] but not all [67, 68] studies. Moreover, Lima et al. [69] did not find a difference in Cu erythrocyte concentrations between obese children and normal weight controls. Laitinen et al. [70] studied a Finnish pediatric cohort documenting a negative correlation between serum Cu and HDL-cholesterol, with obesity not affecting this relationship. Interestingly, serum antioxidant capacity owing to ceruloplasmin (Cp) failure was strongly associated with pediatric NAFLD-related damage. Further, the study by Nobili et al. shows that low Cu and Cp levels are associated with a higher NAS score reflecting hepatic damage. The authors suggested that Cp may serve as an additional noninvasive marker for NAFLD, in particular for NASH, ballooning, and inflammation. There are several hypotheses supporting a role of Cp as an indicator of liver dysfunction: first, there are striking histological similarities between the hypoceruloplasminemia in Wilson disease and severe NAFLD. In NAFLD, a decreased hepatocellular Cp synthesis capacity secondary to liver dysfunction may—as delineated above for a deranged iron metabolism—reflect higher susceptibility to oxidative stress directed to the hepatocyte, which could be related to misfolded proteins and hence ballooning in NASH [71]. In addition, an excessive consumption of fructose, which parallels the increasing prevalence of obesity worldwide, is a well-recognized risk factor for NAFLD [72]. Recent studies showed that experimentally increased fructose intake recapitulates many of the pathophysiological characteristics of the metabolic syndrome in humans including NAFLD [73]. Further, Song et al. [74] suggested that high fructose-induced NAFLD may be due, in part, to inadequate dietary Cu related to impaired duodenum Cu transporter 1 absorption in rats. Thereby, dietary marginal Cu deficiency and fructose feeding would contribute to liver dysfunction and increased lipid accumulation, potentially mediated by iron overload. In keeping with this, building upon knowledge that iron and Cu metabolism are closely linked [75], Cu availability has been shown to contribute to iron perturbations in adults with NAFLD. Further, reduced Cu availability was suggestive of inducing increased iron stores via decreased ferroportin-1 expression and ceruloplasmin ferroxidase activity thus blocking liver iron export in Cu deficient adults [76].

Thus, since the Cu-dependent ferroxidase ceruloplasmin facilitates the release of iron from hepatic cells [77], low concentrations may lead to iron retention and may thereby augment oxidative stress [71]. In contrast to these adult data, markers of iron status were apparently normal in a pediatric NAFLD cohort, while Cp levels appeared disarranged. The discrepancy between adult and these pediatric data was suggested to be explained by an augmented ferroxidase activity in the presence of lower Cp in children as compared to adults [71]. However, considering the paucity of pediatric studies concentrating on the link between NAFLD and changes in serum Cu levels, long-term investigations of pediatric NAFLD cohorts with follow-up into adulthood will be required to clarify the contribution of Cu and ceruloplasmin in NAFLD.

## 7. Summary

Distinct changes of iron and Cu homeostasis are observed in pediatric obesity and NAFLD. Obese children without NAFLD are mainly prone to develop iron deficiency which is related to visceral AT inflammation and consecutively impaired iron uptake. Increased iron requirements due to physical growth likely lead to the manifestation of iron deficiency or even anemia. In NAFLD subjects, hepatic iron deposition has been reported in a manner similar to adult DIOS. Since available data also suggest that Cu and ceruloplasmin may also be involved in NAFLD pathogenesis, it will be an important agenda of research to elucidate details of the interaction of iron and Cu with oxidative stress, insulin resistance, and histological damage in longitudinal studies.

## References

[1] Corey K. E., Kaplan L. M. (2014). Obesity and liver disease: the epidemic of the twenty-first century. *Clinics in Liver Disease*.

[2] Schwimmer J. B., Deutsch R., Kahen T., Lavine J. E., Stanley C., Behling C. (2006). Prevalence of fatty liver in children and adolescents. *Pediatrics*.

[3] Feldstein A. E., Charatcharoenwitthaya P., Treeprasertsuk S., Benson J. T., Enders F. B., Angulo P. (2009). The natural history of non-alcoholic fatty liver disease in children: a follow-up study for up to 20 years. *Gut*.

[4] Schwimmer J. B., Behling C., Newbury R. (2005). Histopathology of pediatric nonalcoholic fatty liver disease. *Hepatology*.

[5] Vajro P., Lenta S., Socha P. (2012). Diagnosis of nonalcoholic fatty liver disease in children and adolescents: position paper of the ESPGHAN hepatology committee. *Journal of Pediatric Gastroenterology and Nutrition*.

[6] Jacobs P., Finch C. A. (1971). Iron for erythropoiesis. *Blood*.

[7] Hallberg L. (1981). Bioavailability of dietary iron in man. *Annual Review of Nutrition*.

[8] Mims M. P., Prchal J. T. (2005). Divalent metal transporter 1. *Hematology*.

[9] Ganz T. (2005). Cellular iron: ferroportin is the only way out. *Cell Metabolism*.

[10] Korolnek T., Hamza I. (2014). Like iron in the blood of the people: the requirement for heme trafficking in iron metabolism. *Drug Metabolism and Transport*.

[11] Ponka P., Beaumont C., Richardson D. R. (1998). Function and regulation of transferrin and ferritin. *Seminars in Hematology*.

[12] Petrak J., Vyoral D. (2005). Hephaestin—a ferroxidase of cellular iron export. *International Journal of Biochemistry and Cell Biology*.

[13] Newman R., Schneider C., Sutherland R., Vodinelich L., Greaves M. (1982). The transferrin receptor. *Trends in Biochemical Sciences*.

[14] Kohgo Y., Ikuta K., Ohtake T., Torimoto Y., Kato J. (2008). Body iron metabolism and pathophysiology of iron overload. *International Journal of Hematology*.

[15] Ganz T. (2003). Hepcidin, a key regulator of iron metabolism and mediator of anemia of inflammation. *Blood*.

[16] Nemeth E., Tuttle M. S., Powelson J. (2004). Hepcidin regulates cellular iron efflux by binding to ferroportin and inducing its internalization. *Science*.

[17] Bekri S., Gual P., Anty R. (2006). Increased adipose tissue expression of hepcidin in severe obesity is independent from diabetes and NASH. *Gastroenterology*.

[18] Kulaksiz H., Fein E., Redecker P., Stremmel W., Adler G., Cetin Y. (2008). Pancreatic beta-cells express hepcidin, an iron-uptake regulatory peptide. *Journal of Endocrinology*.

[19] Lee P. L., Beutler E. (2009). Regulation of hepcidin and iron-overload disease. *Annual Review of Pathology: Mechanisms of Disease*.

[20] Chung B., Matak P., McKie A. T., Sharp P. (2007). Leptin increases the expression of the iron regulatory hormone hepcidin in HuH7 human hepatoma cells. *Journal of Nutrition*.

[21] Nemeth E., Rivera S., Gabayan V. (2004). IL-6 mediates hypoferremia of inflammation by inducing the synthesis of the iron regulatory hormone hepcidin. *The Journal of Clinical Investigation*.

[22] Gehrke S. G., Kulaksiz H., Herrmann T. (2003). Expression of hepcidin in hereditary hemochromatosis: evidence for a regulation in response to the serum transferrin saturation and to non-transferrin-bound iron. *Blood*.

[23] Nairz M., Haschka D., Demetz E., Weiss G. (2014). Iron at the interface of immunity and infection. *Frontiers in Pharmacology*.

[24] Aeberli I., Kaspar M., Zimmermann M. B. (2007). Dietary intake and physical activity of normal weight and overweight 6- to 14-year-old Swiss children. *Swiss Medical Weekly*.

[25] Moschonis G., Chrousos G. P., Lionis C., Mougios V., Manios Y., Healthy Growth Study Group (2012). Association of total body and visceral fat mass with iron deficiency in preadolescents: the Healthy Growth Study. *British Journal of Nutrition*.

[26] Nead K. G., Halterman J. S., Kaczorowski J. M., Auinger P., Weitzman M. (2004). Overweight children and adolescents: a risk group for iron deficiency. *Pediatrics*.

[27] Cepeda-Lopez A. C., Osendarp S. J. M., Melse-Boonstra A. (2011). Sharply higher rates of iron deficiency in obese Mexican women and children are predicted by obesity-related inflammation rather than by differences in dietary iron intake. *The American Journal of Clinical Nutrition*.

[28] Pinhas-Hamiel O., Newfield R. S., Koren I., Agmon A., Lilos P., Phillip M. (2003). Greater prevalence of iron deficiency in overweight and obese children and adolescents. *International Journal of Obesity*.

[29] Eftekhari M. H., Mozaffari-Khosravi H., Shidfar F. (2009). The relationship between BMI and iron status in iron-deficient adolescent Iranian girls. *Public Health Nutrition*.

[30] Ferrari M., Mistura L., Patterson E. (2011). Evaluation of iron status in European adolescents through biochemical iron indicators: the HELENA Study. *European Journal of Clinical Nutrition*.

[31] Baumgartner J., Smuts C. M., Aeberli I., Malan L., Tjalsma H., Zimmermann M. B. (2013). Overweight impairs efficacy of iron supplementation in iron-deficient South African children: a randomized controlled intervention. *International Journal of Obesity*.

[32] Amato A., Santoro N., Calabrò P. (2010). Effect of body mass index reduction on serum hepcidin levels and iron status in obese children. *International Journal of Obesity*.

[33] Gong L., Yuan F., Teng J. (2014). Weight loss, inflammatory markers, and improvements of iron status in overweight and obese children. *Journal of Pediatrics*.

[34] Aeberli I., Hurrell R. F., Zimmermann M. B. (2009). Overweight children have higher circulating hepcidin concentrations and lower iron status but have dietary iron intakes and bioavailability comparable with normal weight children. *International Journal of Obesity*.

[35] Mujica-Coopman M. F., Brito A., López de Romaña D., Pizarro F., Olivares M. (2014). Body mass index, iron absorption and iron status in childbearing age women. *Journal of Trace Elements in Medicine and Biology*.

[36] del Giudice E. M., Santoro N., Amato A. (2009). Hepcidin in obese children as a potential mediator of the association between obesity and iron deficiency. *The Journal of Clinical Endocrinology and Metabolism*.

[37] Falzacappa M. V. V., Spasic M. V., Kessler R., Stolte J., Hentze M. W., Muckenthaler M. U. (2007). STAT3 mediates hepatic hepcidin expression and its inflammatory stimulation. *Blood*.

[38] Tussing-Humphreys L., Frayn K. N., Smith S. R. (2011). Subcutaneous adipose tissue from obese and lean adults does not release hepcidin *in vivo*. *TheScientificWorldJournal*.

[39] Ganz T., Nemeth E. (2009). Iron sequestration and anemia of inflammation. *Seminars in Hematology*.

[40] Manco M., Alisi A., Real J.-M. F. (2011). Early interplay of intra-hepatic iron and insulin resistance in children with non-alcoholic fatty liver disease. *Journal of Hepatology*.

[41] Demircioğlu F., Görünmez G., Dağıstan E. (2014). Serum hepcidin levels and iron metabolism in obese children with and without fatty liver: case-control study. *European Journal of Pediatrics*.

[42] Piperno A. (1998). Classification and diagnosis of Iron overload. *Haematologica*.

[43] Aigner E., Feldman A., Datz C. (2014). Obesity as an emerging risk factor for iron deficiency. *Nutrients*.

[44] Valenti L., Fracanzani A. L., Bugianesi E. (2010). HFE genotype, parenchymal iron accumulation, and liver fibrosis in patients with nonalcoholic fatty liver disease. *Gastroenterology*.

[45] Aigner E., Hinz C., Steiner K. (2010). Iron stores, liver transaminase levels and metabolic risk in healthy teenagers. *European Journal of Clinical Investigation*.

[46] Tan T. C. H., Crawford D. H. G., Jaskowski L. A. (2013). Excess iron modulates endoplasmic reticulum stress-associated pathways in a mouse model of alcohol and high-fat diet-induced liver injury. *Laboratory Investigation*.

[47] van de Wier B., Balk J. M., Haenen G. R. M. M. (2013). Elevated citrate levels in non-alcoholic fatty liver disease: the potential of citrate to promote radical production. *FEBS Letters*.

[48] Moya D., Baker S. S., Liu W. (2014). Novel pathway for iron deficiency in pediatric non-alcoholic steatohepatitis (1042.1). *The FASEB Journal*.

[49] Brunt E. M. (2010). Pathology of nonalcoholic fatty liver disease. *Nature Reviews Gastroenterology and Hepatology*.

[50] Tessari P., Coracina A., Cosma A., Tiengo A. (2009). Hepatic lipid metabolism and non-alcoholic fatty liver disease. *Nutrition, Metabolism and Cardiovascular Diseases*.

[51] Halliwell B., Gutteridge J. M. C. (1984). Oxygen toxicity, oxygen radicals, transition metals and disease. *Biochemical Journal*.

[52] Halliwell B., Gutteridge J. M. C. (1990). Role of free radicals and catalytic metal ions in human disease: an overview. *Methods in Enzymology*.

[53] Sorrentino P., D'Angelo S., Ferbo U., Micheli P., Bracigliano A., Vecchione R. (2009). Liver iron excess in patients with hepatocellular carcinoma developed on non-alcoholic steato-hepatitis. *Journal of Hepatology*.

[54] Datz C., Felder T. K., Niederseer D., Aigner E. (2013). Iron homeostasis in the metabolic syndrome. *European Journal of Clinical Investigation*.

[55] Weismüller T. J., Kirchner G. I., Scherer M. N. (2011). Serum ferritin concentration and transferrin saturation before liver transplantation predict decreased long-term recipient survival. *Hepatology (Baltimore, Md)*.

[56] Walker N. M., Stuart K. A., Ryan R. J. (2010). Serum ferritin concentration predicts mortality in patients awaiting liver transplantation. *Hepatology*.

[57] Scheiber I., Dringen R., Mercer J. F. B., Sigel A., Sigel H., Sigel R. K. O. (2013). Copper: effects of deficiency and overload. *Interrelations between Essential Metal Ions and Human Diseases*.

[58] Al-Othman A. A., Rosenstein F., Lei K. Y. (1992). Copper deficiency alters plasma pool size, percent composition and concentration of lipoprotein components in rats. *Journal of Nutrition*.

[59] Aigner E., Strasser M., Haufe H. (2010). A role for low hepatic copper concentrations in nonalcoholic fatty liver disease. *The American Journal of Gastroenterology*.

[60] Klevay L. M. (2011). Is the Western diet adequate in copper?. *Journal of Trace Elements in Medicine and Biology*.

[61] Al-Othman A. A., Rosenstein F., Lei K. Y. (1993). Copper deficiency increases in vivo hepatic synthesis of fatty acids, triacylglycerols, and phospholipids in rats. *Proceedings of the Society for Experimental Biology and Medicine*.

[62] Prohaska J. R., Geissler J., Brokate B., Broderius M. (2003). Copper,zinc-superoxide dismutase protein but not mRNA is lower in copper-deficient mice and mice lacking the copper chaperone for superoxide dismutase. *Experimental Biology and Medicine*.

[63] Schuschke D. A., Adeagbo A. S. O., Patibandla P. K., Egbuhuzo U., Fernandez-Botran R., Johnson W. T. (2009). Cyclooxygenase-2 is upregulated in copper-deficient rats. *Inflammation*.

[64] Wei Y., Rector R. S., Thyfault J. P., Ibdah J. A. (2008). Nonalcoholic fatty liver disease and mitochondrial dysfunction. *World Journal of Gastroenterology*.

[65] Cayir Y., Cayir A., Turan M. I. (2014). Antioxidant status in blood of obese children: the relation between trace elements, paraoxonase, and arylesterase values. *Biological Trace Element Research*.

[66] Azab S. F. A., Saleh S. H., Elsaeed W. F., Elshafie M. A., Sherief L. M., Esh A. M. H. (2014). Serum trace elements in obese Egyptian children: a case-control study. *Italian Journal of Pediatrics*.

[67] Tascilar M. E., Ozgen I. T., Abaci A., Serdar M., Aykut O. (2011). Trace elements in obese Turkish children. *Biological Trace Element Research*.

[68] Suliburska J., Cofta S., Gajewska E. (2013). The evaluation of selected serum mineral concentrations and their association with insulin resistance in obese adolescents. *European Review for Medical and Pharmacological Sciences*.

[69] Lima S. C. V. C., Arrais R. F., Sales C. H. (2006). Assessment of copper and lipid profile in obese children and adolescents. *Biological Trace Element Research*.

[70] Laitinen R., Vuori E., Viikari J. (1989). Serum zinc and copper: associations with cholestrol and triglyceride levels in children and adolescents. Cardiovascular risk in young Finns. *Journal of the American College of Nutrition*.

[71] Nobili V., Siotto M., Bedogni G. (2013). Levels of serum ceruloplasmin associate with pediatric nonalcoholic fatty liver disease. *Journal of Pediatric Gastroenterology and Nutrition*.

[72] Spruss A., Bergheim I. (2009). Dietary fructose and intestinal barrier: potential risk factor in the pathogenesis of nonalcoholic fatty liver disease. *Journal of Nutritional Biochemistry*.

[73] Yilmaz Y. (2012). Review article: is non-alcoholic fatty liver disease a spectrum, or are steatosis and non-alcoholic steatohepatitis distinct conditions?. *Alimentary Pharmacology & Therapeutics*.

[74] Song M., Schuschke D. A., Zhou Z. (2012). High fructose feeding induces copper deficiency in Sprague-Dawley rats: a novel mechanism for obesity related fatty liver. *Journal of Hepatology*.

[75] Sharp P. (2004). The molecular basis of copper and iron interactions. *Proceedings of the Nutrition Society*.

[76] Aigner E., Theurl I., Haufe H. (2008). Copper availability contributes to iron perturbations in human nonalcoholic fatty liver disease. *Gastroenterology*.

[77] Young S. P., Fahmy M., Golding S. (1997). Ceruloplasmin, transferrin and apotransferrin facilitate iron release from human liver cells. *FEBS Letters*.

